# Comparison of the efficacy of intravitreal Anti-VEGF versus intravitreal dexamethasone implant in treatment resistant diabetic Macular Edema

**DOI:** 10.1186/s12886-023-02831-6

**Published:** 2023-03-13

**Authors:** Hakan Koc, Atilla Alpay, Suat Hayri Ugurbas

**Affiliations:** 1 Faculty of Medicine, Department of Ophthalmology, Giresun University, Giresun, Turkey; 2 Faculty of Medicine, Department of Ophthalmology, Zonguldak Bulent Ecevit University, Zonguldak, Turkey

**Keywords:** Dexamethasone implant, Ranibizumab, Aflibercept, Diabetic macular edema

## Abstract

**Purpose:**

Comparison of the efficacy of monthly anti-VEGF versus dexamethasone (DEX) implant in patients with diabetic macular edema (DME) whose macular edema persists despite three doses of anti-VEGF therapy.

**Materials and methods:**

This retrospective study included 94 eyes of 94 patients with central macular thickness (CMT) > 300 μm despite previously receiving three doses of anti-VGEF (aflibercept or ranibizumab) injections between January 2014 and January 2019. The patients were divided into four groups. The first and second groups were the patients who received three more doses of initial anti-VGEF treatment after the initial anti-VGEF treatment. The third and fourth groups were patients switched to intravitreal dexamethasone implants. Patients were followed up every month for six months after the injection. The primary outcome measures were best-corrected visual acuity (BCVA), central macular thickness (CMT), and intraocular pressure (IOP) at six months.

**Results:**

The mean age of the patients included in the study was 64.64 ± 7; there were 58 men (61.7%) and 36 women (38.3%). There was no statistically significant difference between the groups regarding age, stage of retinopathy, and lens status. When CMT, BCVA, and IOP were assessed among the four groups at the end of the sixth month, no statistical difference between the groups was found. There was no need for medical intervention despite the statistically significant increase in IOP at the end of the sixth month compared to the third month in the dexamethasone implanted groups. In contrast to the decrease in CMT, which was statistically significant in all four groups at the end of the sixth month compared to the third month, the increase in BCVA was not statistically significant in any of the four groups at the end of the sixth month.

**Conclusion:**

According to the results of our study, there is no superiority between continuing with existing anti-VEGF or switching to a dexamethasone implant after three doses of anti-VEGF.

## Introduction

Diabetic retinopathy (DRP) is a specific microvascular complication of Diabetes Mellitus (DM) and is the leading cause of vision loss worldwide in middle-aged and economically active people [1]. The leading cause of visual loss in diabetic patients is diabetic macular edema (DME). Inflammation, angiogenesis, and oxidative stress are involved in the pathogenesis of DME, which is mostly caused by interleukin (IL)-6, -8, and cytokines such as monocyte chemotactic protein and vascular endothelial growth factor (VEGF) [2]. Intravitreal anti-VEGF injections have improved visual acuity and reduced retinal thickness in DME eyes [3]–[4]. Nonetheless, macular edema persisted in 32–66% of eyes treated with injections for at least six months, and visual acuity declined in general [3]. Therefore, additional treatments are needed for eyes that do not sufficiently respond to anti-VEGF medication.

Intravitreal steroids are utilized in the treatment of DME because they inhibit VEGF secretion and vascular permeability while stabilizing the lysosomal membranes and blood-retinal barrier [5]. Dexamethasone implant (Ozurdex; Allergan Inc., Irvine, CA) is a slow-release dexamethasone delivery system designed for intravitreal administration that was recently introduced as a treatment option for DME [6]. Studies have also revealed that intravitreal Dexamethasone may be beneficial for DME patients who do not respond well to anti-VEGF therapy since corticosteroids act on distinct targets than anti-VEGF medicines [7].

## Materials and methods

### Study population

The study was conducted in accordance with the Helsinki Declaration’s tenets, and the local ethics committee’s approval was required before the study could begin. Each patient provided an informed consent form before intravitreal injection. Between January 2014 and January 2019, 94 eyes of 94 patients with NPDR or early PDR with a central macular thickness (CMT) greater than 300 microns despite three doses of anti-VEGF therapy for persistent diabetic macular edema were assessed retrospectively and included in the study. When both eyes passed the inclusion criteria, only the right eye was included since there was a propensity for the outcome measurements from the two eyes of the same participant to be positively associated. Before receiving the injection, each patient was informed of the procedure’s benefits, dangers, and potential side effects and granted informed consent. 18 years and above with Type 2 DM who had previously received three monthly anti-VEGF injections but still had CMT values above 300 μm were enrolled in the study.

### Criteria for exclusion

- High-risk PDR.
- Pregnancy.
- Uncontrolled hypertension.
- Retinal vascular disease, Age-related macular degeneration, presence of epiretinal membrane in OCT.
- Uveitis, Glaucoma.
- History of pars plana vitrectomy.
- Severe cataracts.
- History of Yag laser capsulotomy within six months.
- Patients who underwent laser photocoagulation were not included in the study.

### Study design

The patients were divided into four groups. Group 1, patients who received an additional three doses of Aflibercept after three doses of Aflibercept treatment (AFL + AFL); group 2, patients who received an additional three doses of ranibizumab after three doses of ranibizumab treatment (RAN + RAN), group 3, patients who underwent intravitreal Dexamethasone after three doses of Aflibercept treatment (AFL + DEX), group 4, patients who received intravitreal Dexamethasone (RAN + DEX) after three doses of ranibizumab treatment were patients. Detailed ophthalmologic examinations of the patients were performed. The best corrected visual acuity (BCVA) with the Snellen chart, anterior segment examinations with a biomicroscope, intraocular pressures (IOP) with a Goldmann applanation tonometer, and posterior segment examinations using 90D non-contact lenses after pupil dilatation was performed. CMT was measured by OCT (Heidelberg Engineering, Heidelberg, Germany). For the analysis between the groups, the values obtained in the sixth month after the injection in each of the four groups were calculated and evaluated. All of the patients included in the study with the diagnosis of DME were evaluated with Fundus Fluorescein Angiography (FFA), and macular ischemia was ruled out.

### Treatment protocol

Before the injection, the conjunctiva and skin were cleansed with 5% and 10% povidone-iodine, respectively. In blepharos, the sterile eye cover was concealed and worn. The upper temporal region was chosen as the injection site, and topical proparacaine was used prior to the injection. Anti-VEGF and dexamethasone implants were injected into the vitreous at a distance of 4 mm from the limbus in phakic patients and 3.5 mm from the limbus in pseudophakic patients using their respective injector systems. We assessed post-injection light detection and IOP. After the injection, all patients received topical moxifloxacin 0.5% prophylaxis four times per day for one week and were called for infection throughout the first postoperative week. All patients were reviewed at one week, one month, two months, three months, four months, five months, and six months after injection. At each review visits, all patients underwent comprehensive ophthalmologic tests. The BCVA, CMT, and IOP were documented. Patients who missed their monthly follow-up appointments were eliminated from the study.

### Statistical analysis

SPSS 19.0 (SPSS Inc., Chicago, IL, USA) was used for statistical analysis. The Shapiro-Wilk test was used to determine whether numerical variables fit the normal distribution. Descriptive statistics were expressed as numbers and percentages for categorical data, while mean, standard deviation, and median (minimum-maximum) were used for numerical variables. The Chi-square test was used to examine the categorical variable differences between the groups. A Kruskal-Wallis analysis of variance was used to compare the four groups in terms of numerical variables. The measurement values between the two dependent groups were compared using the Wilcoxon-marked sequence(s) test. p < 0.05 was considered significant.

## Results

The demographic characteristics of the patients included in the evaluation are summarized in Table 1.

There was no statistically significant difference between the four groups regarding age, stage of retinopathy, laterality, or lens status. (phakic, pseudophakic). (p > 0.05)

When the mean visual acuity, mean central macular thickness and mean intraocular pressure were evaluated after three doses of anti-VEGF treatment, there was no statistically significant difference between the four groups. (p > 0.05) Table 2.

### İntragroup analysis

Visual acuity after three doses of anti-VEGF treatment (3rd month) and six doses of anti-VEGF treatment or third month (6th month) of DEX implant treatment was evaluated using intragroup analysis. There was no statistically significant difference between the mean visual acuity in the third month and the mean visual acuity in the sixth month in any of the four groups. (p > 0.05)

The mean central macular thickness after three doses of anti-VEGF treatment (3rd month) and six doses of anti-VEGF treatment or the third month (6th month) of DEX implant treatment was evaluated using intragroup analysis. There was a statistically significant difference between the mean central macular thickness in the third month and the mean central macular thickness in the six month in all four groups.(p<0.05)

The mean intraocular pressure after three doses of anti-VEGF treatment (3rd month) and six doses of anti-VEGF treatment or the third month (6th month) of DEX implant treatment was evaluated using intragroup analysis. There was a statistically significant difference between the mean intraocular pressure in the third month and the mean intraocular pressure in the six month in groups that switched to Dexamethasone implant.(p<0.05) None of these individuals, however, required medical or surgical treatment.

### Intergroup analysis

For the analysis between the groups, the values ​​obtained in the sixth month after the injection in each of the four groups were calculated and evaluated.

When the values obtained in mean visual acuity, mean central macular thickness, and mean intraocular pressure were compared between the four groups, there was no statistically significant difference between the groups at six months. (p < 0.05) Table 4.

**Fig. 1:**
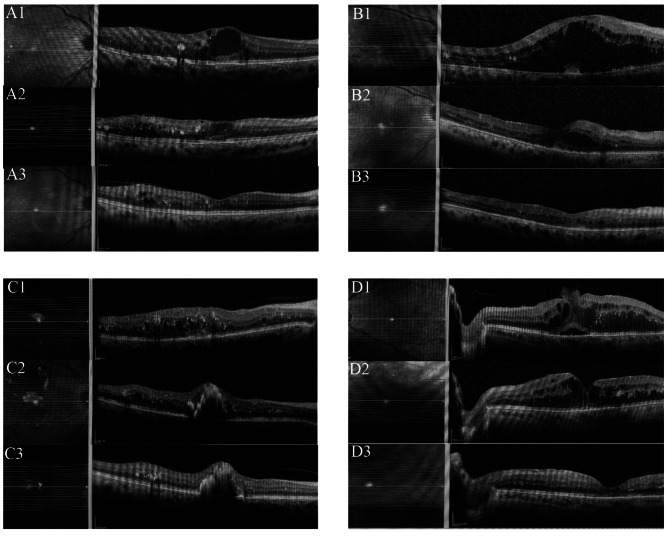
OCT images of patients A1. OCT image before treatment A2. OCT image after 3 doses of Aflibercept A3. OCT image after 6 doses of Aflibercept B1. OCT image before treatment B2. OCT image after 3 doses of ranibizumab B3. OCT image after 6 doses of ranibizumab C1. OCT image before treatment C2. OCT image after 3 doses of Aflibercept C3. OCT image at 3months of Dexamethasone implant D1.OCT image before treatment D2. OCT image after 3 doses of ranibizumab D3. OCT image at 3 months of Dexamethasone implant

OCT images of some patients in the groups are shown in Fig. 1.

## Discussion

Diabetic macular edema (DME) is a common consequence of diabetic retinopathy (DRP) and the leading cause of vision loss among persons of working age in developed nations [8]–[9]. Numerous studies indicate a correlation between VEGF level and retinopathy activity and suggest that VEGF is the principal angiogenic agent responsible for the development of diabetic retinopathy and maculopathy [10]–[11]. With the discovery of VEGF’s function in the pathophysiology of DME, anti-VEGF medicines have become the standard treatment.

In the “Study of Safety and Efficacy of ranibizumab in Diabetic Macular Edema” (RESOLVE) and “Study of ranibizumab for Diabetic Macular Edema” (RISE and RIDE) investigations, patients who received injections of 0.3 mg and 0.5 mg of ranibizumab were compared to the control group. After 12 months, the groups receiving intravitreal ranibizumab had a significant improvement in visual acuity and a significant decrease in central macular thickness [12]. In the study titled “An exploratory study of the safety, tolerability, and bioactivity of a single intravitreal injection of vascular endothelial growth factor Trap-Eye in patients with diabetic macular edema,“ the efficacy and safety of intravitreal Aflibercept were assessed. Six weeks following the injection, the central macular thickness and mean letter gain in visual acuity decreased [13]. Compared to other studies, the central macular thickness in the AFL + AFL group and the RAN + RAN group decreased statistically significantly in the sixth month compared to the third month. However, the increase in visual acuity was not statistically significant in the sixth month compared to the third month, unlike other studies.

Corticosteroids stabilize lysosomal membranes and the blood-retina barrier while diminishing VEGF secretion and vascular permeability. For this reason, corticosteroid medications are utilized to treat DME [5]. In a study examining the changes in the amounts of inflammatory and angiogenic cytokines in the humoral aqueous after intravitreal injection of triamcinolone and bevacizumab in patients with DME, IL6, inducible protein 10, monocyte chemoattractant protein, platelet-derived growth factor AA, and VEGF were observed to be significantly decreased in eyes injected with triamcinolone, only VEGF has been shown to be reduced in eyes injected with bevacizumab [14]. In another study, it was stated that corticosteroids in DME may be more meaningful than anti-VEGF therapy, which is effective only on the part of the inflammatory cascade [15]. Intravitreal corticosteroids in DME; It can be considered if anti-VEGFs are contraindicated, if there is poor compliance with repetitive anti-VEGF applications and if there is resistance to anti-VEGFs [16]. In the study of Vujosevic et al., Dexamethasone is effective in cases of increased foveal autofluorescence [17]. In the study of Vural et al., it was concluded that dexamethasone implant responds better than anti-VEGF treatment in DME cases with subretinal fluid [18]. In the MEAD study, 0.7 mg dexamethasone was assigned at random to 0.35 mg dexamethasone and placebo groups. It was determined that the intravitreal dexamethasone group had a greater decrease in central macular thickness and letter gain. In addition, it was observed that nearly one-third of dexamethasone-treated patients required treatment for intraocular hypertension [19].

In the study conducted by Totan et al., after intravitreal dexamethasone administration to patients who received three doses of 2.5 mg intravitreal bevacizumab at 6-week intervals and whose central macular thickness was greater than 275 microns, central macular thickness decreased significantly in the first, third, and sixth months. Additionally, the average visual acuity and intraocular pressure increased significantly in the first and third month [20]. In the study of Zhioua et al., intravitreal dexamethasone was administered to 12 patients whose central macular thickness continued to be 300 microns and above, despite six consecutive months of ranibizumab, and they found that dexamethasone was influential on the central macular thickness and visual acuity [21]. Simsek et al. applied the DEX implant to patients with central macular thickness greater than 300 microns despite at least six doses of ranibizumab treatment and showed that the intravitreal DEX implant significantly improved visual acuity and central macular thickness values in patients with DME resistant to anti-VEGF therapy [22]. Yorgun et al. showed that intravitreal dexamethasone implantation resulted in significant improvement in BCVA and reduction in CMT in patients with persistent DME who did not respond to three consecutive injections of ranibizumab [23].

In the study by Maturi et al. (Protocol U), the group that continued to receive 0.3 mg ranibizumab after three doses of 0.3 mg ranibizumab was compared to the group that received a Dexamethasone implant. Although the decrease in mean central macular thickness and increase in mean intraocular pressure were more pronounced in the group that was switched to dexamethasone after 24 weeks, there was no difference in mean visual acuity [7].

In a meta-analysis study by Khan et al., it was reported that the DEX implant applied to patients with refractory DME despite the use of anti-VEGF agents improves vision and may be a treatment alternative for patients with DME who have a low response to anti-VEGF agents [24].

Ozata et al. demonstrated that dexamethasone implantation raised BCVA and decreased CMT in DME patients refractory to sequential intravitreal ranibizumab therapy over the initial three months. They reported that intravitreal dexamethasone implantation could be a viable alternative treatment for DME that is resistant [25].

Busch et al. observed that in patients with central macular thickness > 300 microns after three doses of ranibizumab treatment, the group that switched to a Dexamethasone implant had better visual and anatomical results than the group that continued with ranibizumab [26].

In our study, at the conclusion of the sixth month, we compared the groups that continued with three doses of anti-VEGF after three doses of anti-VEGF treatment and the groups that were switched to intravitreal dexamethasone implants. The decrease in central macular thickness, the rise in intraocular pressure, and the rise in visual acuity were not statistically significant. Despite the statistically significant increase in intraocular pressure between the third and sixth months in the groups implanted with dexamethasone, the patients did not require anti-glaucoma medication.

### Strengths and limitations

This study has disadvantages such as being retrospective, having a small number of patients in the groups, being unable to evaluate inflammatory markers, and having an average follow-up period of 6 months. However, the absence of a similar study conducted on four different groups is also important for our study. Our study should be supported by prospective, long-term follow-up and further studies with large patient groups. New molecules with fewer side effects and longer acting are needed for better therapeutic results.

## Conclusion

According to the results of this study, there is no superiority between continuing with the current anti-VEGF treatment or switching to a dexamethasone implant after three doses of anti-VEGF, as there is no statistically significant difference between the mean visual acuity and central macular thickness at six months. According to the results of our research, after three doses of anti-VEGF (3rd month), to apply for a medication change (to switch to dexamethasone implant treatment) should be selected according to the patient (presence of glaucoma, compliance with treatment, etc.) and cost. Dexamethasone implant has an advantage over anti-VEGF in that it needs fewer injections, and its cost is cheaper than three doses of anti-VEGF. In addition, while the rise in intraocular pressure was statistically significant in the groups that moved to dexamethasone, it is also important for the transition to dexamethasone because this condition does not need medical or surgical treatment.

**Table 1:** Demographic characteristics of the data

	AFL + AFL	RAN + RAN	AFL + DEX	RAN + DEX	P value
Age	62.68 ± 6.9	64.74 ± 7.6	64.27 ± 8.0	66.9 ± 5.6	0.466
Stage of Retinopathy (NPDR/PDR)	20/5	22/4	17/5	15/6	0.705
Laterality R/L	13/12	12/14	13/9	12/9	0.736
Lens Status (Phakic/Psodophakic)	15/10	16/10	11/11	10/11	0.226

**Table 2:** Comparison of treatment groups after 3 doses of anti-VEGF (3rd month) in terms of evaluation parameters

	AFL + AFL	RAN + RAN	AFL + DEX	RAN + DEX	P Value
Visual Acuity (LogMAR)	0.59 ± 0.26	0.64 ± 0.32	0.8 ± 0.4	0.85 ± 0.43	0.260
Central Macular Thickness (µm)	403.6 ± 70.4	411.5 ± 84	413.6 ± 88.1	418.1 ± 88	0.964
Intraocular Pressure (mmHg)	15.3 ± 2.7	15.4 ± 2.9	14.7 ± 2.9	16.2 ± 3.2	0.392

**Table 3:** Comparison of intragroup mean visual acuity, mean central macular thickness and mean intraocular pressure values ​​after 3 anti-VEGF (3 months) and 6 anti-VEGF post or DEX 3 months (6 months)

	AFL + AFL	RAN + RAN	AFL + DEX	RAN + DEX
Visual Acuity (LogMAR) after 3 anti-VEGF (3rd month)	0.59 ± 0.26	0.64 ± 0.32	0.8 ± 0.4	0.85 ± 0.43
6 Anti-VEGF or DEX 3rd month Average Visual Acuity (LogMAR) (6th month)	0.49 ± 0.22	0.57 ± 0.38	0.62 ± 0.38	0.80 ± 0.43
P value 3rd month*6th month Visual Acuity (LogMAR)	0.132	0.284	0.064	0.704
Central Macular Thickness (µm) (3rd month) after 3 anti-VEGF	403.6 ± 70.4	411.5 ± 84	413.6 ± 88.1	418.1 ± 88
6 Post-anti-VEGF or DEX 3rd month Central Macular Thickness (µm) (6th month)	291.6 ± 54.4	293.1 ± 79.5	320 ± 103.8	332.5 ± 96.8
P value 3rd month*6th month Central Macular Thickness (µm)	**0.000**	**0.000**	**0.001**	**0.000**
Intraocular Pressure (mmHg) (3rd month) after 3 anti-VEGF	15.3 ± 2.7	15.4 ± 2.9	14.7 ± 2.9	16.2 ± 3.2
6 Post-anti-VEGF or DEX 3rd month Intraocular Pressure (mmHg) (6th month)	15.9 ± 3.5	15.59 ± 4	15.9 ± 3	18.1 ± 3.8
P value 3rd month*6th month Intraocular Pressure (mmHg)	0.542	0.834	**0.013**	**0.027**

**Table 4:** Evaluation of mean visual acuity, mean central macular thickness and mean intraocular pressure between groups at 6 months after injection

	AFL + AFL	RAN + RAN	AFL + DEX	RAN + DEX	P value
Average Visual Acuity LogMAR (6th month)	0.49 ± 0.22	0.57 ± 0.38	0.62 ± 0.38	0.80 ± 0.43	0.159
Central Macular Thickness (µm) (6th month)	291.6 ± 54.4	293.1 ± 79.5	320 ± 103.8	332.5 ± 96.8	0.295
Intraocular Pressure (mmHg) (6th month)	15.9 ± 3.5	15.59 ± 4	15.9 ± 3	18.1 ± 3.8	0.109

## Data Availability

The datasets used and/or analyzed during the current study are available from the corresponding author upon reasonable request.
